# *SPEF1* and *SPEF2* as potential biomarkers in bladder cancer: Insights from a comprehensive bioinformatic analysis

**DOI:** 10.14440/bladder.2024.0071

**Published:** 2025-04-11

**Authors:** Mohamed A. A. A. Hegazi, Fabio Pasqualini, Maurizio Chiriva-Internati, Gianluigi Taverna, Fabio Grizzi

**Affiliations:** 1 Department of Immunology and Inflammation, IRCCS Humanitas Research Hospital, Rozzano, Milan 20089, Italy; 2 Departments of Gastroenterology, Hepatology and Nutrition, Division of Internal Medicine, The University of Texas MD Anderson Cancer Center, Houston, Texas 77030, United States; 3 Department of Urology, Humanitas Mater Domini, Castellanza, Varese 21100, Italy; 4 Department of Biomedical Sciences, Humanitas University, Pieve Emanuele, Milan 20090, Italy

**Keywords:** Bladder, Cancer, Cancer-testis antigens, *SPEF1*, *SPEF2*, Biomarkers, Survival

## Abstract

**Background:**

Bladder cancer (BLCA) remains a prevalent and complex malignancy characterized by significant heterogeneity. Treatment strategies are diverse, based on patient characteristics and cancer stage. Early identification of biomarkers is crucial for improving diagnosis, staging, and treatment planning. These biomarkers offer valuable insights into lesion features, tumor differentiation, and disease progression, thereby playing a pivotal role in the personalized management of BLCA.

**Objective:**

This study investigated the expression of cancer-testis antigens *SPEF1* and *SPEF2* in BLCA using comprehensive bioinformatic analyses to assess their potential as biomarkers.

**Methods:**

The UALCAN database, based on The Cancer Genome Atlas datasets, was employed to compare *SPEF1* and *SPEF2* expression levels in normal bladder tissues and BLCA samples. In addition, the Kaplan–Meier Plotter, OncoDB, and TIMER 2.0 platforms were utilized to evaluate the prognostic and immunotherapeutic relevance of these antigens.

**Results:**

The findings suggest that *SPEF1* and *SPEF2* are integral to various biological processes driving BLCA onset and progression. Both genes appear to facilitate BLCA cell progression and migration, contributing to poor prognosis through specific pathways and by altering tumor microenvironment. Notably, *SPEF1* expression was significantly upregulated in BLCA tissues compared to normal tissues. Conversely, higher *SPEF2* expression was associated with longer overall survival and positively correlated with immunotherapeutic targets.

**Conclusion:**

Although these results were derived from *in silico* analyses, they offer insights into the potential roles of *SPEF1* and *SPEF2* as biomarkers. Further studies are warranted to validate these biomarkers in retrospective patient cohorts to establish their clinical utility.

## 1. Introduction

Globally, bladder cancer (BLCA) poses a significant health challenge due to its high incidence and mortality rates. BLCA represents one of the most common cancers worldwide, particularly in males, where it was the sixth most frequently diagnosed cancer in 2022, with 471,293 new cases reported.^1^ Moreover, it was the ninth leading cause of cancer-related deaths in this group, accounting for over 165,672 fatalities. BLCA is a heterogeneous malignancy with diverse treatment alternatives, depending on patient characteristics and cancer stage.^2^,^3^ It is broadly classified into non-muscle-invasive BLCA (NMIBLCA) and muscle-invasive BLCA (MIBLCA), with MIBLCA typically requiring more aggressive treatment. Its primary treatment involves transurethral resection of the bladder tumor, followed by intravesical therapy with agents such as mitomycin C or Bacillus Calmette–Guérin (BCG) to reduce the risk of recurrence. In contrast, MIBLCA, which includes stages T2, T3, and T4, makes up roughly 20% of BLCA cases and typically entails more aggressive treatment.

Despite advancements in therapy, management of BLCA remains complicated and necessitates a multidisciplinary approach. The primary diagnostic methods for BLCA are cystoscopy and cytology, which are considered the gold standards for the detection of BLCA. Additional diagnostic tools include biopsy and imaging modalities, such as magnetic resonance imaging, computed tomography (CT), positron emission tomography (PET), PET-CT, ultrasonography, and other radiographic techniques. While cystoscopy is effective for detecting BLCA in patients presenting with hematuria, it is invasive, expensive, and often unnecessary, as most hematuria cases are unrelated to BLCA.^4^ Meanwhile, the early detection of biomarkers is essential for improving diagnosis, staging, and treatment planning. These biomarkers provide valuable insights into lesion features, tumor differentiation, and disease progression, significantly contributing to the personalized management of BLCA.

Cancer-testis antigens (CTAs) represent a family of tumor antigens with significant immunogenicity and unique expression patterns in humans.^5^ CTAs are a large family of proteins exclusively expressed in the testis, placenta, and certain types of malignant tumors, where they regulate critical processes during tumorigenesis and progression.^6^ They are categorized as immunogenic tumor-associated antigens and deemed optimal targets for the design of therapeutic cancer vaccines.^7^ To date, over 200 CTAs have been identified and documented in the Cancer-Testis database (www.cta.lncc.br), with more than 100 gene families exhibiting high expression in malignant tumors. Most genes within the same CTA subfamily are located in adjacent positions on the chromosomes, and their encoded proteins generally share similar domains and structural characteristics. In tumor tissues, members of the CTA subfamily are frequently co-expressed and perform similar cellular functions.

In recent years, several CTAs associated with BLCA have been reported, including the melanoma-associated antigen A (MAGE-A) family,^8^ testis-expressed 10,^9^ sperm-associated antigen 6,^10^ and CTA 1B.^11^ These CTA may contribute to BLCA progression and offer promising targets for immunotherapy strategies. For instance, MAGEA9 has been identified not only as a prognostic and diagnostic marker but also as an immunotherapeutic target. Nishiyama *et al*.^12^ used autologous dendritic cells pulsed with the MAGEA3 peptide as an immunotherapy option.

The current study was the first of its kind to explore the potential clinical role of *SPEF1* and *SPEF2* in BLCA, building upon previous research that identified these proteins in other cancers and potentially premalignant lesions. Recently, we have reported the expression of the CTA *SPEF1* in a rare ciliated foregut cyst of the common hepatic duct and shown an upregulation of *SPEF1* mRNA in various cancers across different stages, ethnicities, and genders compared to normal tissues.^13^ SPEF1 has been reported as a multifunctional protein that interacts with cytoskeletal structures, including microtubules, actin filaments, and focal adhesions in epithelia, binds to microtubules and actin-based structures, and regulates cell migration and epithelial cell polarity.^14^-^17^ It has been shown that *SPEF1* has evolutionary orthologs in a wide range of species, including mammals, other vertebrates, *Drosophila*, and protozoans with motile cilia or flagella. A second homolog of the gene, *SPEF2*, is also present in several species, suggesting that these genes are part of a novel gene family.^14^ Interestingly, mRNA encoding SPEF2 has been found in multiple myeloma^18^ and nasopharyngeal carcinoma.^19^ SPEF2, which plays an important role in the differentiation and function of ciliated cells, has also been included as a member of the CTA family (www.cta.lncc.br/index.php). The present study aimed to explore the expression of *SPEF1* and *SPEF2* in BLCA through comprehensive bioinformatic analysis to gain new insights into their potential as BLCA biomarkers.

## 2. Methods

### 2.1. UALCAN analysis

UALCAN (The University of Alabama at Birmingham cancer data analysis portal; https://ualcan.path.uab.edu) is a primary public resource utilized for exploring gene expression data from The Cancer Genome Atlas (TCGA).^20^ It allows for the analysis of relative gene expression across cancerous and normal samples, as well as within different cancer subgroups based on clinicopathological characteristics. In this study, we utilized UALCAN to assess the expression of *SPEF1* and *SPEF2* in BLCA. In addition, we compared the methylation levels of *SPEF1* and *SPEF2* promoters across BLCA and normal tissues. The beta value, ranging from 0 (unmethylated) to 1 (fully methylated), represents the degree of DNA methylation. Diverse cut-off beta values were applied to distinguish between hypermethylation (beta value: 0.7 – 0.5) and hypomethylation (beta value: 0.3 – 0.25). Statistical significance was set at *p*<0.05.^20^-^22^

### 2.2. Kaplan–Meier plotter survival analysis

The Kaplan–Meier Plotter (https://kmplot.com/analysis/index.php?p=background) was used for survival analysis on BLCA datasets by categorizing patients into low and high expression groups for target genes.^23^,^24^ The analysis included overall survival (OS) and relapse-free survival (RFS) using a Jetset probe. Gene symbols and Affymetrix IDs used were SPEF1 (1444397_at) and SPEF2 (1446747_at).

### 2.3. Promoter methylation analysis

OncoDB (https://oncodb.org/index.html) is a comprehensive cancer database that provides valuable insights into genetic alterations across various cancers.^25^ It facilitates in-depth exploration of oncogenes and supports researchers in understanding molecular profiles of cancer. In this study, OncoDB was utilized to perform survival analyses of *SPEF1* and *SPEF2* expression and methylation in BLCA patients.

### 2.4. Expression of *SPEF1* and *SPEF2* in relation to immune cells, cancer-associated fibroblasts (CAFs), and endothelial cells (ECs) in BLCA

The single-sample gene-set enrichment analysis algorithm was performed to compare differences in immune cell infiltration between high and low *SPEF1* and *SPEF2* expression groups. In addition, the impact of somatic cell copy number alterations of the *SPEF1* and *SPEF2* on immune cell infiltration levels was assessed using the Tumor Immune Estimation Resource 2.0 (TIMER 2.0, https://cistrome.shinyapps.io/timer/). Six immune infiltrating cell types were used the B cell, macrophage, dendritic cell, neutrophil cell, cluster of differentiation 4-positive T cells (CD4^+^ T cell), and cluster of differentiation 8-positive T cells (CD8^+^ T cell). To explore the relationship between immune checkpoints (programmed death ligand 1, cytotoxic T-lymphocyte-associated protein 4, lymphocyte activation gene 3, programmed cell death 1, and T cell immunoreceptor with immunoglobulin and immunoreceptor tyrosine-based inhibitory motif domain) and *SPEF1*, we evaluated the expression of these immune checkpoints in the high and low *SPEF1* expression groups using the Wilcoxon test.

## 3. Results

**Figure 1:**
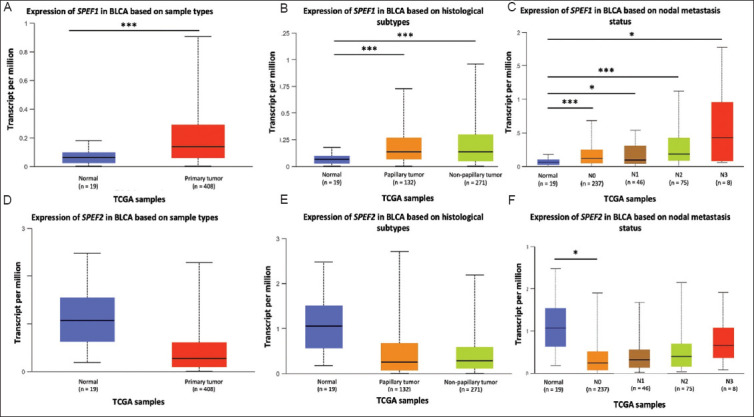
*SPEF1* and *SPEF2* expression in bladder cancer. The expression levels were categorized in terms of (A and D) sample types, (B and E) histological subtypes, and (C and F) nodal metastasis status. Notes: *p **< 0**.05 and ***p**<**0.001, versus normal tissues.

*SPEF1* was significantly upregulated in BLCA tissues compared to normal tissues (*p*=1.550 × 10^−15^, Figure 1A). In contrast, *SPEF2* showed higher expression in normal tissues than in BLCA tissues, although this difference did not reach statistical significance (*p*=0.218, Figure 1D). When BLCA samples were categorized by histological subtypes, *SPEF1* expression remained significantly higher in normal tissues compared to papillary tumors (*p*=9.850 × 10^−7^, Figure 1B) and non-papillary tumors (*p*=5.710 × 10^−13^). In contrast, no significant differences in expression were observed for *SPEF2* across these groups (Figure 1E).

Multiple comparisons revealed that the expression of *SPEF1* across molecular subtypes of BLCA had statistically significant differences (data not shown): normal versus neuronal (*p*=0.0157), normal versus basal squamous (*p*=1.87 × 10^−7^), normal versus luminal (*p*=0.046), normal versus luminal infiltrated (*p*=4.188 × 10^−5^), normal versus luminal papillary (*p*=2.962 × 10^−7^). The expression of *SPEF2* based on molecular subtypes of BLCA^26^ was found to be significantly different in several comparisons: normal versus neuronal (*p*=4.544 × 10^−2^), normal versus luminal (*p*=2.143 × 10^−6^), neuronal versus basal squamous (*p*=2.368 × 10^−2^), neuronal versus luminal (*p*=3.219 × 10^−3^), neuronal versus luminal infiltrated (*p*=1.672 × 10^−2^), neuronal versus luminal papillary (*p*=2.646 × 10^−2^), basal squamous versus luminal (*p*=2.041 × 10^−10^), luminal versus luminal infiltrated (*p*=4.381 × 10^−8^), and luminal versus luminal papillary (*p*=9.237 × 10^−8^).

The expression of *SPEF1* in BLCA samples varied significantly based on nodal status (Figure 1C). Significant differences were observed between normal tissues and N0 (*p*=1.670 × 10^−9^), N1 (*p*=0.0373), N2 (*p*=1.720 × 10^−5^), and N3 (*p*=0.0468) samples. *SPEF2* expression was found to be statistically significant when comparing normal samples to N0 status (*p*=0.0439, Figure 1F).

Furthermore, the expression levels of *SPEF1* and *SPEF2* were significantly correlated with the subjects’ weight (data not shown). Specifically, *SPEF1* expression showed significant differences between normal-weight individuals (body mass index [BMI] ≥18.5 and <25) and those classified as obese (BMI ≥30 and <40, *p*=0.0425), as well as between normal-weight and extremely obese individuals (BMI ≥40, *p*=0.0146). In addition, a significant difference was observed between overweight individuals (BMI ≥25 and <30) and those classified as extremely obese (*p*=0.0201). *SPEF2* expression demonstrated significant differences between overweight individuals (BMI ≥25 and <30) and those classified as extremely obese (BMI ≥40, *p*=0.0443).

Age-related differences in the expression levels of *SPEF1* and *SPEF2* were also observed (data not shown). *SPEF1* expression showed statistically significant differences between subjects aged 21 – 40 years and those aged 41 – 60 years (*p*=7.410 × 10^−4^), 61 – 80 years (*p*=6.750 × 10^−8^), and 81 – 100 years (*p*=6.960 × 10^−3^). Meanwhile, significant differences in *SPEF2* expression were noted between subjects aged 41 – 60 years and those aged 61 – 80 years (*p*=0.0150), as well as between subjects aged 41 – 60 years and 81 – 100 years (*p*=0.0115). *SPEF1* expression was also found to differ significantly between non-smokers and reformed smokers who had quit for more than 15 years (*p*=0.0217, data not shown). Both *SPEF1* and *SPEF2* expression were found to differ significantly between tumor protein p53 (TP53) mutant and TP53 non-mutant samples, with *p*-value being 0.0052 and 0.0045, respectively.

**Figure 2:**
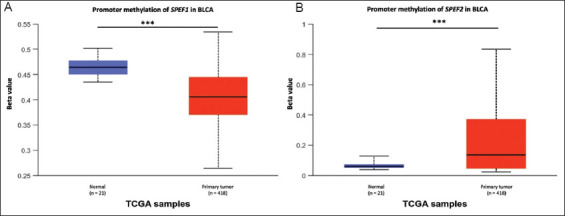
Promoter analysis of *SPEF1* and *SPEF2* in bladder cancer. Promoter methylation levels of (A) *SPEF1* and (B) *SPEF2* in bladder cancer. Note: ***p<0.001, versus normal tissues. Abbreviation: TCGA: The cancer genomic atlas.

The methylation of DNA, a key epigenetic mechanism, plays a critical role in gene expression regulation. To better understand the regulatory dynamics of *SPEF1* and *SPEF2*, we investigated promoter methylation levels in BLCA patients. Our analysis uncovered distinct patterns of promoter methylation between the two genes. Notably, the *SPEF1* promoter exhibited hypomethylation (*p*=1.330 × 10^−15^), indicating potential gene derepression (Figure 2A). Conversely, the *SPEF2* promoter showed hypermethylation (*p*=7.970 × 10^−7^), suggesting possible gene suppression (Figure 2B). Interestingly, these methylation patterns correlated with various clinicopathological features, including tumor histology, patient age, gender, weight, smoking status, TP53 mutation status, and tumor, node and metastasis stage.

**Figure 3:**
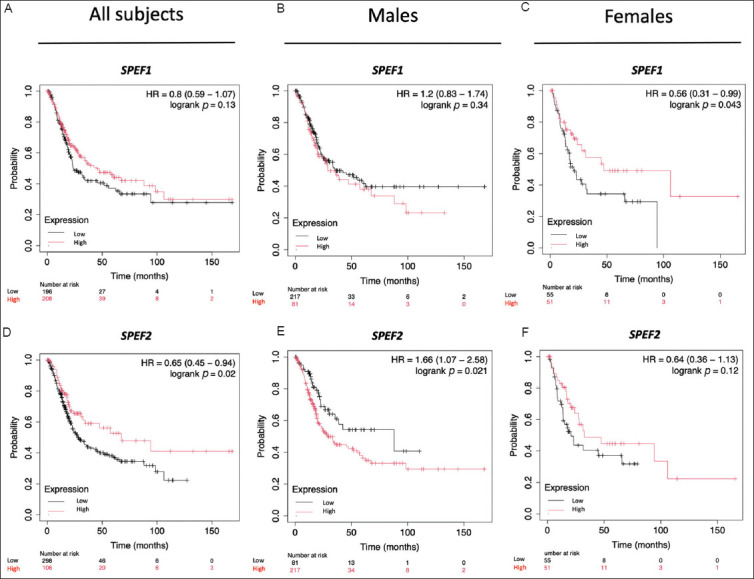
Correlation of *SPEF1* and *SPEF2* expression with overall survival in bladder cancer patients. The overall survival is analyzed for (A and D) the entire cohort, and stratified by gender: (B and E) males and (C and F) females. Abbreviation: HR: Hazard ratio.

**Figure 4:**
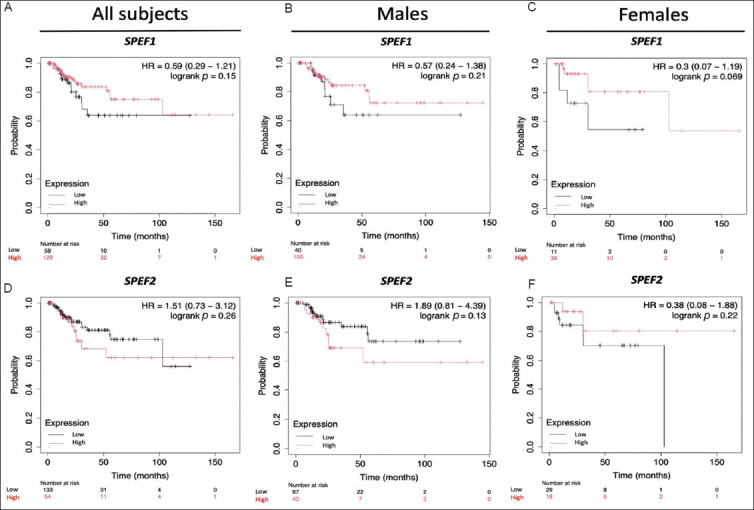
Correlation of *SPEF1* and *SPEF2* expression with relapse-free survival in bladder cancer patients. RFS is analyzed for (A and D) the entire cohort, and stratified by gender: (B and E) males and (C and F) females. Abbreviation: HR: Hazard ratio.

Next, we examined the roles of *SPEF1* and *SPEF2* in OS and RFS among BLCA patients (Figure 3). The findings revealed that *SPEF1* expression was not significantly correlated with OS, whereas higher *SPEF2* expression was associated with longer OS (*p*=0.02). Interestingly, when patients were analyzed in terms of gender, higher *SPEF1* expression was linked to longer OS in female patients (*p*=0.043) but exerted no significant impact on OS in males. Conversely, higher *SPEF2* expression was associated with shorter OS in males (*p*=0.021) but had no effect in females. These patterns are opposite to that observed in the overall population. Given the growing recognition of sex chromosomes as critical factors influencing health and disease beyond their role in biological sex determination, these findings warrant further investigation. On the other hand, neither *SPEF1* nor *SPEF2* expression demonstrated a statistically significant association with RFS (Figure 4). However, it is worth noting that higher *SPEF1* expression and lower *SPEF2* expression tended to be associated with longer RFS. Notably, no gender-specific associations with RFS were identified.

**Figure 5:**
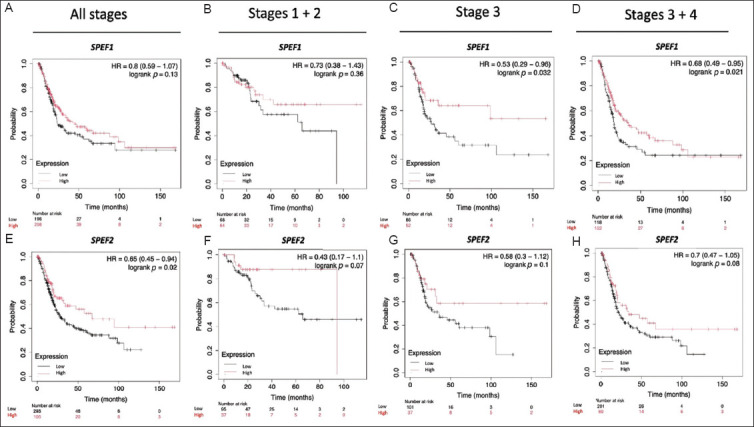
Correlation of *SPEF1* and *SPEF2* expression with overall survival in bladder cancer patients. The overall survival is evaluated for (A and E) the entire cohort, and stratified by BLCA stages: (B and F) stages 1+2, (C and G) stage 3, and (D and H) stages 3+4. Abbreviation: HR: Hazard ratio.

We further analyzed the relationship between *SPEF1* and *SPEF2* expression levels and OS across different BLCA stages (Figure 5). Higher *SPEF1* expression was significantly associated with longer OS in stage 3 (*p*=0.021) and in combined stages 3 and 4 (*p*=0.031). Conversely, no significant association was observed between *SPEF2* expression levels, OS, and BLCA stages. In various cancer types, a high tumor mutational burden is linked to longer survival following treatment with immune checkpoint inhibitors.^25^ However, we observed no correlation between *SPEF1* expression, mutational burden status, and patient survival. Conversely, patients with high *SPEF2* expression and a high tumor mutational burden exhibited shorter OS (*p*=0.0023, Hazard ratio [HR]: 2.17, 95% CI: 1.3 – 3.62), (data not shown).

Using OncoDB,^25^ we assessed the prognostic value of *SPEF1* and *SPEF2* expression and methylation in BLCA. Kaplan–Meier survival analyses were conducted to evaluate their predictive value for patient outcomes. While no significant association was observed between *SPEF2* methylation patterns and OS in BLCA, *SPEF1* methylation patterns showed a significant correlation with OS when stratified by pathological M stage (*p*=0.0021) and smoking status (*p*=0.000063).

**Figure 6:**
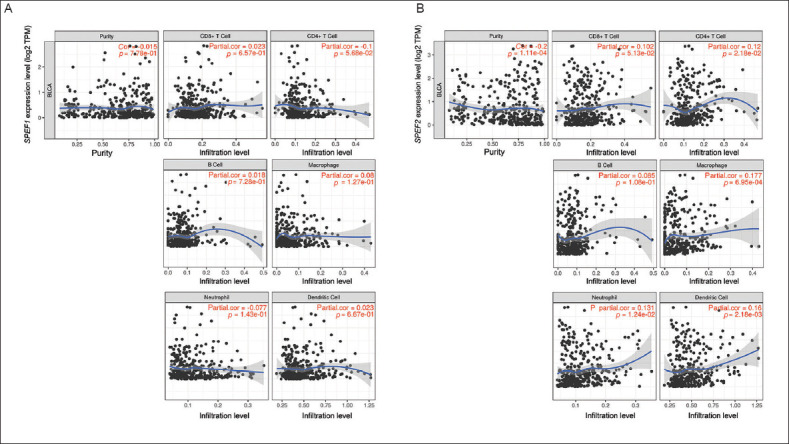
*SPEF1* and *SPEF2* expression levels and immune cell infiltration. Correlation of (A) *SPEF1* and (B) *SPEF2* expression levels with immune cell infiltration in bladder cancer.

To investigate the correlation of *SPEF1* and *SPEF2* expression levels and immune cell infiltration in BLCA, TIMER 2.0 was employed (Figure 6). The mRNA expression levels of both genes were analyzed in relation to tumor purity. *SPEF1* expression showed a negative correlation with CD4^+^ T cell infiltration (Rho = -0.1, *p*=5.680 × 10^−2^) and a negative correlation with neutrophil infiltration (Rho = −0.077, *p*=0.143). In contrast, *SPEF2* expression was positively correlated with the infiltration of B cells (Rho = 0.085, *p*=0.108), macrophages (Rho = 0.177, *p*=6.950 x 10^−4^), myeloid dendritic cells (Rho = 0.1160, *p*=2.180 × 10^−3^), neutrophils (Rho = 0.131, *p*=1.240 × 10^−2^) and with CD4^+^ T cell infiltration (Rho = 0.120, *p*=2.180 × 10^−2^).

**Figure 7:**
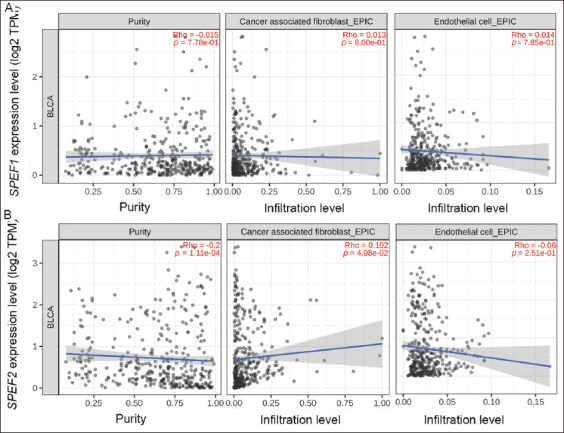
*SPEF1* and *SPEF2* expression and cancer-associated fibroblast and endothelial cell infiltration. Correlation of (A) *SPEF1* and (B) *SPEF2* expression levels with cancer-associated fibroblast and endothelial cell infiltration in bladder cancer.

**Figure 8:**
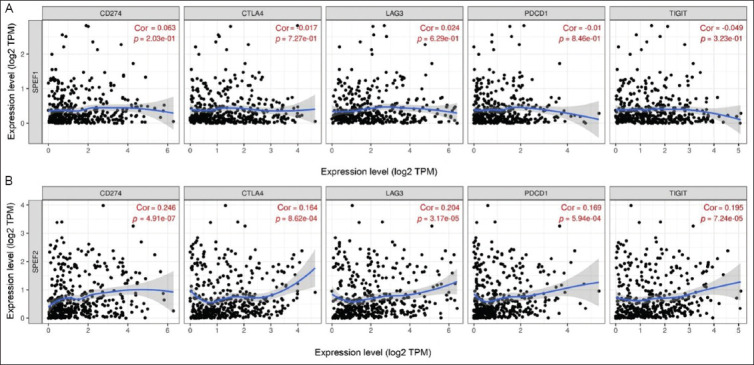
*SPEF1* and *SPEF2* expression and immune checkpoints inhibitors. Correlation of (A) *SPEF1* and (B) *SPEF2* expression with the degree of immune checkpoints infiltration of programmed death ligand 1 (CD274), cytotoxic T-lymphocyte-associated protein 4, lymphocyte activation gene 3, programmed cell death 1, and T cell immunoreceptor with immunoglobulin and immunoreceptor tyrosine-based inhibitory motif domain. Abbreviation: Cor: Correlation.

When examining the correlation of *SPEF1* and *SPEF2* expression levels with the infiltration of cancer-CAFs and ECs in BLCA, we found that *SPEF1* expression bored no significant correlation with the infiltration of either cell type. In contrast, *SPEF2* expression was positively correlated with CAF infiltration (Rho = 0.102, *p*=0.0498), but no correlation was observed with EC infiltration (Figure 7). When assessing the correlation of *SPEF1* and *SPEF2* expression levels with various key immune checkpoints (programmed death ligand 1, cytotoxic T-lymphocyte-associated protein 4, lymphocyte activation gene 3, programmed cell death 1, and T cell immunoreceptor with immunoglobulin and immunoreceptor tyrosine-based inhibitory motif domain), strong positive correlations were found between *SPEF2* expression and these immune checkpoints (Figure 8).

## 4. Discussion

Urinary system cancers are among the most common malignancies across the globe.^1^ Among them, renal cancer, BLCA, and prostate cancer are most prevalent and pose a substantial threat to human health and the quality of life. Prostate cancer is the most common malignant tumor of the male urinary system. Meanwhile, BLCA is one of the most frequently diagnosed malignancies, with a strong sex-related disparity – men exhibit a significantly higher incidence than women. Urothelial BLCA is a heterogeneous epithelial malignancy that typically presents as an exophytic tumor confined to the mucosa or lamina propria. However, approximately 25% of patients are diagnosed with MIBLCA or metastatic disease and associated with a worse prognosis.^26^ The current treatment strategies for both MIBLCA and NMIBLCA primarily rely on clinical and histopathological characteristics. However, a substantial proportion of patients fails to respond to guideline-based therapies, highlighting the urgent need for predictive biomarkers that can optimize therapeutic approaches while minimizing adverse effects. While cystoscopy remains the gold standard for BLCA diagnosis and monitoring, it is invasive, costly, and poses risks such as infection, pain, and occasional hematuria.^27^ Advances in tumor genetics, pharmacology, and high-throughput genomics technologies have facilitated the development of novel biomarkers and targeted therapies. Ideally, these advancements aim to identify biomarkers and therapeutic targets with high specificity and efficacy, being highly expressed in malignant tissues while remaining absent or minimally expressed in normal tissues. However, despite significant progress, no single biomarker has been proven as a reliable predictor of BLCA recurrence or prognosis.^28^ Many currently available biomarkers suffer from high false-negative rates, underscoring the need for more accurate and effective diagnostic tools.

CTAs, a class of tumor-associated antigens, have emerged as promising targets in cancer research due to their unique expression profiles. CTAs are typically found in embryonic stem cells and testicular germ cells, with minimal to no expression in most somatic tissues. However, they are aberrantly expressed in various cancers, including BLCA. Among these, chromosome X-encoded CT antigens antigens are highly expressed in BLCA.^28^,^29^ An early study identified increased expression of *MAGEA1 (*21% of cases) and *MAGEA3 (*35% of cases) in primary transitional cell carcinoma of the bladder.^30^ Further research linked elevated expression of several *MAGE* genes with advanced disease stages, with *MAGEA9* and *MAGEA4* being particularly associated with metastasis and recurrence. This highlights *MAGEA9* as a potential prognostic and diagnostic marker, as well as a promising immunotherapeutic target.^31^ Building on these findings, the present study utilized comprehensive bioinformatic analyses to investigate the expression of *SPEF1* and *SPEF2* in BLCA, aiming to uncover their potential as novel biomarkers.

Both *SPEF1* and *SPEF2* have been linked to clinicopathological features of BLCA, suggesting their potential roles as novel biomarkers for the disease. Their proteins are associated with key biological processes relevant to gametogenesis and carcinogenesis, including responses to hyperoxia, cilium movement, embryo implantation, extracellular matrix organization, GTPase activity, and alpha-tubulin binding. Our results provided promising evidence for *SPEF1* and *SPEF2* as potential BLCA biomarkers. The BLCA microenvironment is known to consist of diverse components, including tumor cells, immune cells, and stromal cells, interacting through immune checkpoint molecules, cytokines, and chemokines.^32^,^33^ Recently, Li *et al*.^34^ have highlighted the heterogeneity of the BLCA microenvironment, identifying biologically distinct subtypes that can predict prognosis and responses to anti-programmed death-ligand 1 therapy. Our analysis revealed that *SPEF1* expression was positively correlated with CD4^+^ T cell infiltration, while negatively associated with neutrophil infiltration. In contrast, *SPEF2* expression was positively correlated with infiltration of CAF and all immune cell subtypes tested in our study, except CD4^+^ T cells, which showed a negative correlation, and CD8^+^ T cells. It is well-established that T cells (particularly cytotoxic T cells and helper T cells), natural killer cells, and dendritic cells are crucial for recognizing and eliminating tumor cells in immunotherapy. In addition, macrophages, B cells, and regulatory T cells play important roles in immune responses and tumor microenvironment, making them valuable targets for immunomodulation. Research also shows that BCG therapy induces inflammatory responses involving various immune cell subsets that eliminate cancer cells either through direct cytotoxicity or the secretion of toxic compounds, such as the tumor necrosis factor-inducing ligand.^35^ The immune subsets include CD4^+^ and CD8^+^ lymphocytes, natural killer cells, granulocytes, macrophages, and dendritic cells. While BCG therapy has demonstrated high response rates (55 – 65% for high-risk papillary tumors and 70 – 75% for carcinoma *in situ*), 25 – 45% of patients fail to respond. This highlights the need for a more in-depth understanding of the immunobiology of BCG-induced tumor immunity. Such insights are crucial for tailoring more personalized and effective BCG treatment to specific patients, improving its efficacy, and reducing treatment intolerance. Investigating the role of novel CTAs, such as SPEF1 and SPEF2, in BLCA within the immune system may provide insights into novel immunotherapeutic approaches, such as the use of autologous dendritic cells pulsed with SPEF1 and SPEF2 peptides.

Although TCGA data provide valuable insights into cancer genomics and biomarker discovery, several limitations must be acknowledged. TCGA dataset data are collected from multiple institutions, resulting in variability in sample quality and standardization. In addition, besides its comprehensive molecular data (i.e., gene expression, mutations, copy number alterations), TCGA often lacks detailed clinical information, such as treatment history, therapy response, and longitudinal follow-up data, limiting its ability to correlate molecular alterations with clinical outcomes or therapeutic responses. Furthermore, TCGA data primarily focus on tumor cells, neglecting the complex interactions between the tumor and its microenvironment, which could play a crucial role in cancer progression and therapeutic response. Another limitation of TCGA is that its database is predominantly composed of European and North American populations, raising concerns about the generalizability of the findings to other ethnic or geographic groups. Despite these limitations, *in silico* analysis offers significant advantages by enabling researchers to explore and predict potential therapeutic targets, biomarkers, and drug interactions using large-scale genomic and transcriptomic data. This could accelerate the discovery of new targets for drug development and personalized medicine. Moreover, *in silico* approaches also facilitate the generation and prioritization of hypotheses for further experimental validation.

In summary, our results suggest that SPEF1 and SPEF2 may play a key role in the onset and progression of BLCA, as well as in the regulation of the complex BLCA microenvironment through interactions with various cell types. As potential biomarkers for BLCA, both SPEF1 and SPEF2 require further investigation involving a larger cohort of BLCA patients to assess the expression of these proteins in body fluids, cytological samples, and tissue specimens.

## 5. Conclusion

Several novel biomarkers that capture key tumor and immune features are rapidly emerging. However, a major technical challenge in biomarker research lies in methodological variability, which complicates the prospective validation of different biomarker tests. In this study, we have outlined a pipeline to explore the potential of novel biomarkers for BLCA. While these preliminary findings were derived from *in silico* analyses, they provide a promising foundation for future research. Further investigation should analyze the expression of *SPEF1* and *SPEF2* in a larger cohort of BLCA cases, thereby correlating these results with clinicopathological data, patient follow-up, and therapeutic strategies. A multicenter clinical study will be crucial to obtaining more robust and generalizable results, as it will increase the sample size and enhance external validity. Conducting research across multiple institutions and geographic regions will mitigate potential biases associated with single-center study, ensuring that the findings reflect real-world disease heterogeneity and treatment responses.

## Data Availability

All data presented in the current study were analyzed using the UALCAN database, along with the Kaplan–Meier Plotter, OncoDB, and TIMER 2.0 platforms.
